# CATHETER II: a randomised controlled trial comparing the clinical effectiveness of various washout policies versus no washout policy in preventing catheter-associated complications in adults living with long-term catheters

**DOI:** 10.1136/bmjopen-2024-087203

**Published:** 2024-12-02

**Authors:** Mohamed Abdel-fattah, Muhammad Imran Omar, Diana Johnson, David Cooper, Lynda Constable, Sheela Tripathee, Sara J MacLennan, Konstantinos Dimitropoulos, Suzanne Evans, Hashim Hashim, Mary Kilonzo, James Larcombe, Paul Little, Peter Murchie, Phyo Kyaw Myint, James N'Dow, Catherine Paterson, Karen Powell, Graham Scotland, Nikesh Thiruchelvam, Amanda Young, Seonaidh Cotton, John Norrie, Graeme MacLennan

**Affiliations:** 1 Aberdeen Centre for Women’s Health Research, University of Aberdeen, Aberdeen, UK; 2 Academic Urology Unit, University of Aberdeen, Aberdeen, UK; 3 Centre for Healthcare Randomised Trials, University of Aberdeen, Aberdeen, UK; 4 Department of Urology, Aberdeen Royal Infirmary, NHS Grampian, Aberdeen, UK; 5 Bladder Health UK, Birmingham, UK; 6 Bristol Urological Institute, North Bristol NHS Trust, Bristol, UK; 7 University of Bristol, Bristol, UK; 8 Health Economics Research Unit, Institute of Applied Health Sciences, University of Aberdeen, Aberdeen, UK; 9 NHS Durham Dales Easington and Sedgefield CCG, Sedgefield, UK; 10 Primary Care Research Centre, University of Southampton, Southampton, UK; 11 Academic Primary Care Research Group, Institute of Applied Health Sciences, University of Aberdeen, Aberdeen, UK; 12 Institute of Applied Health Sciences, University of Aberdeen, Aberdeen, UK; 13 School of Nursing, Midwifery and Public Health, University of Canberra, Canberra, Australian Capital Territory, Australia; 14 Cambridge University Hospitals NHS Foundation Trust, Cambridge, UK; 15 Queen's Nursing Institute, London, UK; 16 Centre for Public Health, Queen's University Belfast, Belfast, UK

**Keywords:** clinical trial, urinary tract infections, urology, urinary incontinences

## Abstract

**Objectives:**

Do weekly prophylactic saline or acidic catheter washouts in addition to standard long-term catheter (LTC) care improve the outcomes of adults with LTC compared with standard LTC care only.

**Design:**

Three-arm superiority open-label randomised controlled trial.

**Setting:**

UK community-based study.

**Participants:**

80 adults with LTC (any type/route) ≥28 days in situ with no plans to discontinue and able to self-manage the washouts/study documentation with/without a carer.

**Interventions:**

Randomly allocated (26:27:27) to receive standard LTC care with weekly saline or weekly acidic or no prophylactic washouts for up to 24 months.

**Primary and secondary outcome measures:**

The primary outcome was catheter blockage requiring intervention (per 1000 catheter days). Secondary outcomes were symptomatic catheter-associated urinary tract infection (S-CAUTI) requiring antibiotics, adverse events, participants’ quality of life and day-to-day activities, acceptability and adherence.

**Results:**

Outcomes reported for 25 saline, 27 acidic and 26 control participants. LTC blockages (per 1000 catheter days) requiring treatment were 9.96, 10.53 and 20.92 in the saline, acidic and control groups, respectively. The incident rate ratio (IRR) favours the washout groups (saline 0.65 (97.5% CI 0.24 to 1.77); p=0.33 and acidic 0.59 (97.5% CI 0.22 to 1.63); p=0.25), although not statistically significant. The S-CAUTI rate (per 1000 catheter days) was 3.71, 6.72 and 8.05 in the saline, acidic and control groups, respectively. The IRR favours the saline group (saline 0.40 (97.5% CI 0.20 to 0.80); p=0.003 and acidic 0.98 (97.5% CI 0.54 to 1.78); p=0.93). The trial closed before reaching target recruitment due to reduced research capacity during the COVID-19 pandemic.

**Conclusions:**

Early closure and small sample size limits our ability to provide a definite answer. However, the observed non-statistically significant differences over control are favourable for lower rates of LTC blockages without a concomitant rise in S-CAUTI. The results support a multinational randomised controlled trial of catheter washouts in patients with LTC to ascertain their clinical and cost-effectiveness.

**Trial registration number:**

ISRCTN17116445.

**STRENGTHS AND LIMITATIONS OF THIS STUDY**

- CATHETER II was the largest randomised controlled trial to date investigating prophylactic catheter washouts to prevent blockage.
- A pragmatic trial design was used to evaluate the intervention in real-life practice. Participants were supported to self-manage the intervention to minimise impact on healthcare resources.
- A comprehensive list of outcomes was assessed and relate to patient, healthcare professional, guideline developer and other stakeholder decision making.
- Validated tools were used to assess quality of life, adherence, convenience, satisfaction and impact on daily activities; however, outcome data were patient-reported, and it was not possible to blind participants to the intervention.
- Sample size was limited by early closure of the trial due to difficulty with recruitment during the COVID-19 pandemic. This was primarily a result of reduced research capacity and prioritisation of COVID-19 and cancer-related research in primary and secondary care settings.

## Introduction

 Long-term catheter (LTC) is defined as catheter use for >28 days.^1^ Approximately 90 000 (1 in 700) of the UK population live with LTC (urethral or suprapubic), with a higher prevalence (0.5%) in those aged over 75 years and a mean duration of use of 6 years.^2^,^4^ In a recently published study, Gage *et al*^5^ explored catheter-related service use and costs in patients living with LTC in England. Their findings showed that almost 60% of LTC users were men, 71% participants were >70 years of age and 61% used a urethral catheter. Indications for LTC include intractable urinary incontinence or chronic retention due to spinal cord injury, multiple sclerosis, prostate enlargement and underactive bladder.^6 7^ With an ageing population,^8^ LTC prevalence and LTC-related use of healthcare resources is expected to rise substantially.

Standard LTC care involves a weekly valve and/or leg bag change (by the patient/carer) and a 4–12 weekly catheter change (usually by the clinical team).^9^

Adverse events (AEs) associated with LTC use impact patients’ quality of life (QoL) and are a significant burden on National Health Service (NHS) resources.^10^ AEs can include blockage, symptomatic catheter-associated urinary tract infection (S-CAUTI), urinary leakage, bladder spasms, pain and accidental dislodgement.^5 6^

Blockage is the main AE with an incidence of 20%–70%^11^ or 8.54 per 1000 days of catheter use,^6^ requiring emergency treatment. A Grampian wide audit (Northeast of Scotland) in 2017 showed 11.8 blockages requiring intervention per 1000 catheter days. Rarely, blockage may lead to serious complications of urosepsis or autonomic dysreflexia in patients with spinal cord injury at T6 or above.^11^

Various catheter washout policies are used for the prevention and treatment of catheter blockage including different types of solutions, concentrations, volumes and frequency.^7^ Washouts may work by mechanically flushing debris, dissolving mineral encrustations and/or by antimicrobial effect.^12^ Current guidelines do not recommend prophylactic washouts to prevent blockages suggesting instead more frequent change of the catheter.^1^ The Cochrane review^7^ concluded there is insufficient evidence to define benefits, clinical effectiveness, risks, acceptability and impact on patient’s QoL. They also reported clinical community concerns that catheter washouts may damage bladder mucosa and introduce infection, and recommended a robust randomised controlled trial (RCT) to assess the clinical and cost-effectiveness of prophylactic washouts in adults living with LTC.

CATHETER II hypothesised that weekly prophylactic catheter washouts, in addition to standard LTC care, would result in a ≥25% relative reduction in catheter blockages requiring intervention. Weekly prophylactic normal saline or citric acid washouts were compared in parallel with standard LTC care only. Clinical effectiveness, patient acceptability, satisfaction and safety were evaluated.

## Materials (patients) and methods

CATHETER II was a pragmatic three-arm open-label multicentre superiority RCT with internal pilot and embedded qualitative component. The trial methodology has been published.^13 14^ Summary methods are included in accordance with the Consolidated Standards of Reporting Trials (CONSORT) guidelines.^15^

**Figure 1:**
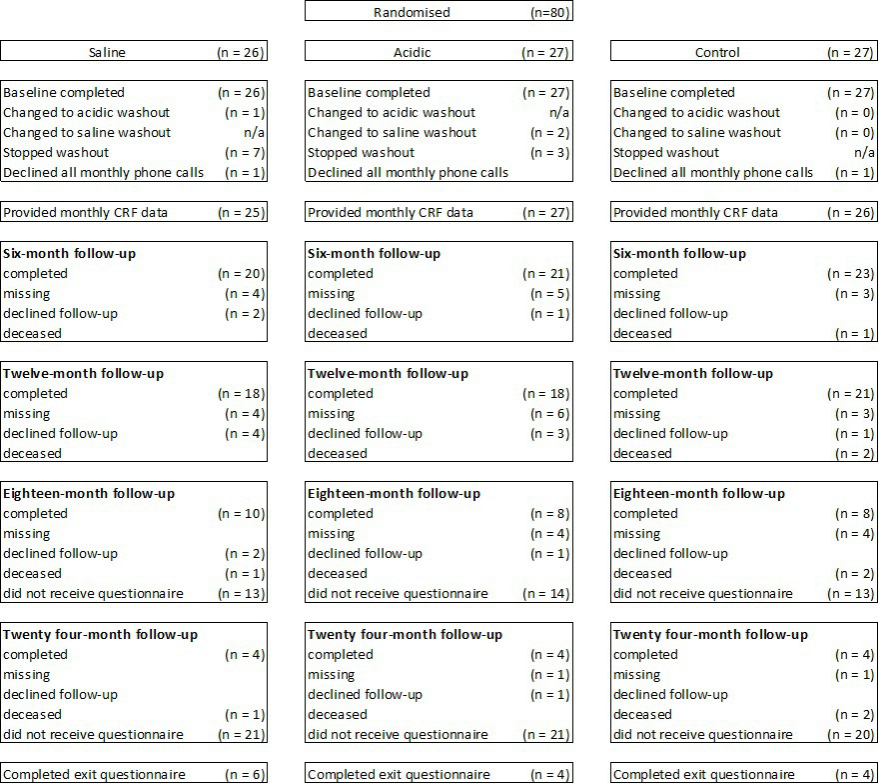
The Consolidated Standards of Reporting Trials diagram.

Participants were recruited from 21 sites in Scotland, England and Wales including general practitioner practices, community/secondary care hospitals and using targeted social media/website advertising. Participants were aged ≥18 years with an LTC (any type/route) in use for at least 28 days with no plan to discontinue. Participants self-managed catheter washouts and completed trial documentation or had a carer to assist them. Patients unable to consent or were pregnant/contemplating pregnancy were not eligible. Also excluded were patients with a spinal cord injury at/above T6, suspected S-CAUTI, visible haematuria, known allergies to the washout solutions, current bladder cancer or bladder stones or who the recruitment team considered unsuitable for other clinical or social reasons (figure 1).

### Interventions

Participants were randomised to receive a policy of:

- weekly prophylactic normal saline (Uro-Tainer NaCl 0.9% 100 mL) catheter washouts plus standard LTC care;
- weekly prophylactic acidic (two sequential applications of 30 mL 3.23% citric acid, Uro-Tainer Twin Suby G) catheter washouts plus standard LTC care;
- standard LTC care only (ie, no prophylactic catheter washouts).

A survey of opinion from relevant stakeholders identified these washouts as the most commonly used washouts in the UK.^13^ They were also the most frequently used interventions identified in the Cochrane systematic review.^7^ A secondary consideration for choice of intervention was product availability within the required timeframe. Due to the choice of comparators and the inclusion of a no prophylactic washout standard care-only arm, it was not possible to blind participants to the intervention. Changes to washout policy during follow-up, including type and frequency, were permitted when clinically necessary. Participants/Carers were trained on best practice washout technique by trained delegated members of staff. To standardise washout delivery, all participants were provided with the same training resources.^13^ Additional training was provided when identified by staff or participant/carer. To minimise breakage of the closed drainage system and the risk of introducing infection, the washouts were administered during the regular change of the catheter bag or valve.

### Outcomes

The primary clinical outcome was catheter blockage requiring intervention up to 24 months post-randomisation expressed as number per 1000 catheter days. An intervention was defined as an unplanned catheter removal or change, or washout performed by the participant or carer, or requiring unplanned visits to/from any healthcare provider, or hospital admission. The primary health economic outcome was the incremental cost per quality-adjusted life year gained with each washout policy compared with standard LTC care only.

Secondary outcomes included S-CAUTI requiring antibiotics, LTC-related other AEs, duration of LTC use, catheter changes (other than for blockage), QoL measures (both generic: EuroQoL 5-Dimension 5-Level (EQ-5D-5L)^16^ and disease-specific: International Consultation on Incontinence Modular Questionnaire-LTC QoL (ICIQ-LTCqol)^17^), adherence, convenience, satisfaction (adapted Treatment Satisfaction Questionnaire for Medication (TSQM)^18^), impact on daily activities (General Self-Efficacy (GSE) Scale^19^ and ICEpop CAPability measure for Adults/Older people (ICE-CAP-A/ICE-CAP-O)^20 21^), discontinuation of LTC and changes to the washout policy.

Outcome data were patient reported. Baseline data were collected prior to randomisation. Follow-up data were collected by monthly telephone call and 6-monthly postal/online questionnaires. Where participant follow-up ended early (discontinuation of LTC or early study closure), an additional EQ-5D-5L questionnaire was collected.

### Sample size

A survey of experts, patients and available literature deemed a 25% reduction in LTC blockage was required for washouts to be considered worthwhile. This implied a reduction from 11.8/1000 days (control) to 8.9. We used the formula from Zhu and Lakkis^22^ to derive the sample size for comparing two negative binomial rates. We needed outcome data from 200 participants per arm for 90% power, assuming two-sided significance of 2.5% (to account for two planned comparisons) and a mean 50/730 days loss to follow-up.

### Randomisation

The local research team randomised participants 1:1:1 to one of three arms using a centralised computer randomisation system (created and administered by Centre for Healthcare Randomised Trials, University of Aberdeen). It was not possible to blind participants or researchers to the allocated arm. Randomisation used a minimisation algorithm with a random element and minimised on the following factors:

Region (NHS Grampian, Aneurin Bevan University Health Board, Cwm Taf University Health Board, CRN Eastern, CRN East Midlands, CRN Thames Valley South Midlands, CRN Yorkshire and Humber, CRN Wessex, CRN Greater Manchester, CRN South London, Central), gender (male vs female), age (<45 years, 45–64 years, 65 years and above), residential status (community vs care home), previous blockages (zero vs 1 or more), previous S-CAUTI (zero vs 1 or more), urine pH (acidic (<5.1) vs normal (5.1–6.7) vs alkaline (>6.7) vs not available).

### Patient and public involvement

Patient and public involvement (PPI) partners were active members of the project management group and Trial Steering Committee. They were involved in all stages of the project including development of the study protocol and study materials and oversight. PPI partners will lead the development of the dissemination for participants and the public.

### Modifications due to impact of COVID-19

Recruitment commenced in December 2019 and was paused in March 2020 due to COVID-19 pandemic regulations. Following extensive efforts, the study team obtained approval from Sponsor and the regulatory authorities and recruitment resumed in September 2020 with protocol modifications to minimise face-to-face contact (postal/telephone consent; telephone collection of baseline data and video consultation training of washout technique). Follow-up continued by post and telephone in line with the original protocol. COVID-19-related protocol adjustments were previously published.^13^

Recovery plans were instigated but the pandemic continued to negatively impact the set-up of research sites and recruitment to the study. In June 2022, the funder elected to terminate the study early, with recruitment ending in August 2022 and follow-up ending in August 2023. The closure plan included completing the qualitative study (published alongside) and providing descriptive analysis only for the health economic measures.

### Statistical methods

Analyses were based on the intention-to-treat principle. Baseline and outcome data are summarised as counts and percentages for categorical data, and as means and SDs for continuous data.

Number of blockages requiring intervention and number of S-CAUTI were summarised as a rate per 1000 catheter days and analysed using a mixed effects negative binomial regression. Fixed effects were included for the intervention (saline or acidic washout) and the minimisation variables gender, age band, previous blockage and previous S-CAUTI. A random effect (intercept) was included for region. An offset was included for the log of catheter duration (in days). Effect sizes are reported as incidence rate ratios (IRR) with 97.5% CIs. QoL measures were analysed with repeated measures mixed effects linear regression. The same fixed effects were included as for the primary outcome. A dummy variable for timepoint was included as participants could report outcomes at 6, 12, 18 and 24 months. Random effects were included for region and participant to adjust for repeated observations on the same participant. The effect sizes are the adjusted mean differences with 97.5% CIs used as an approximation to 95% CIs, as the analysis model estimates the effect size of both saline and acidic washout.

A sensitivity analysis is included making additional adjustments for potential baseline imbalance. This analysis adjusted for: gender (male vs female), age (<45 years, 45–65 years, >65 years), previous blockage (0, 1–3, 4 or more), previous infection (0, 1–3, 4 or more), catheter duration at baseline (<1 year, 1–3 years, >3 years), on washout at baseline (yes vs no), neuropathic bladder (yes vs no), catheter change frequency. Due to the lower recruitment and consequently smaller sample size, the planned subgroup analyses were not conducted. In addition, it was not possible to analyse and report primary (incremental cost per quality-adjusted life year) or secondary (time and travel costs) health economic outcomes. The healthcare resource use are presented as descriptive analyses.

## Results

### Baseline

The mean age of participants in the study was 65 years with those in the control group slightly older and similar numbers of males and females in all three groups (table 1). The urine pH and the number of participants who had blockages requiring treatment or S-CAUTI requiring antibiotics were similar in all three groups at baseline. Catheter change frequencies ranged from every week to every 12 weeks, with the highest number of participants in all three groups changing their LTC every 12 weeks. There was good balance in the patient-reported QoL scores at baseline, although there are small differences between the ICECAP-O scores.

**Table 1:** Baseline data

	Saline washouts (n=26)	Acidic washouts (n=27)	Control (n=27)
Age	64.8 (17.9); (n=26)	62.4 (16.7); (n=27)	67.1 (15.3); (n=27)
Female	14/26 (54%)	12/27 (44%)	14/27 (52%)
Length of time catheterised (years)			
<1	7/26 (27%)	5/27 (19%)	5/27 (19%)
1–3	9/26 (35%)	6/27 (22%)	9/27 (33%)
>3	10/26 (38%)	16/27 (59%)	13/27 (48%)
Neuropathic bladder	8/26 (31%)	9/27 (33%)	11/27 (41%)
Urine pH	6.5 (0.8); (n=24)	6.7 (1.0); (n=25)	6.8 (0.8); (n=25)
Current on washout	3/26 (12%)	6/27 (22%)	6/27 (22%)
Catheter change frequency			
Every week			1/27 (3.7%)
Every 2 weeks			2/27 (7.4%)
Every 3 weeks			1/27 (3.7%)
Every 4 weeks	4/26 (15%)	4/27 (15%)	5/27 (19%)
Every 5 weeks		1/27 (3.7%)	
Every 6 weeks	4/26 (15%)	3/27 (11%)	2/27 (7.4%)
Every 7 weeks		1/27 (3.7%)	1/27 (3.7%)
Every 8 weeks	3/26 (12%)	2/27 (7.4%)	2/27 (7.4%)
Every 10 weeks	2/26 (7.7%)	5/27 (19%)	3/27 (11%)
Every 12 weeks	13/26 (50%)	11/27 (41%)	10/27 (37%)
Blockages requiring treatment (in the prior 6 months)			
0	13/26 (50%)	13/27 (48%)	12/27 (44%)
1–3	8/26 (31%)	9/27 (33%)	7/27 (26%)
4 or more	5/26 (19%)	5/27 (19%)	8/27 (30%)
Median (lower, upper quartile)	0.5; (0, 3)	1; (0, 3)	1; (0, 5)
S-CAUTI requiring antibiotics (in the prior 6 months)			
0	14/26 (54%)	13/27 (48%)	14/27 (52%)
1–3	9/26 (35%)	9/27 (33%)	10/27 (37%)
4 or more	3/26 (12%)	5/27 (19%)	3/27 (11%)
Median (lower, upper quartile)	0; (0, 2)	1; (0, 2)	0; (0, 2)
GSE Scale*	29.1 (9.1); (n=25)	29.4 (5.7); (n=27)	27.8 (7.6); (n=27)
ICIQ-LTCqol function and concern†	18.3 (9.1); (n=26)	17.3 (9.7); (n=26)	19.1 (9.0); (n=27)
ICIQ-LTCqol lifestyle†	6.7 (3.4); (n=24)	8.1 (3.3); (n=27)	7.6 (2.9); (n=27)
EQ-5D-5L‡	0.368 (0.405); (n=25)	0.365 (0.359); (n=26)	0.348 (0.373); (n=27)
ICECAP-A§	0.551 (0.216); (n=10)	0.487 (0.223); (n=11)	0.496 (0.218); (n=9)
ICECAP-O§	0.488 (0.320); (n=15)	0.601 (0.206); (n=14)	0.669 (0.204); (n=15)

Apart from where indicated, the summary statistics for the continuous outcomes are mean, standard deviationSD, and count, while the categorical variables are summarised with count and percentage.

* The general self-efficacy (GSE) sScale assesses ability to cope with daily life. It has 10 questions and scores are between 10 and 40 with higher scores better.

† ICIQ long-term catheterisation quality of life questionnaireLong-Term Catheterisation Quality of Life Questionnaire is a specific quality of life measure. It produces two scores: the function and concern score and the lifestyle score. The function and concern score hasconsists of 10 questions and is on thea scale from 0 to 42. The lifestyle score has 3consists of three questions and is on thea scale 3 from to 15. For both scores, higher scores arevalues indicate worse.

‡ The EQ-5D-5L is a generic quality of life measure. It hasconsists of 5five questions and is on thea scale from −0.594 to 1, where higher scores indicate better quality of life.

§ The ICECAP-A and ICEPOP-O measure capability in adults and older people, respectively. Both haveconsist of 5five questions and are on thea scale from 0 to 1, with higher scores betterindicating better outcomes. The Treatment Satisfaction Questionnairetreatment satisfaction questionnaire assesses satisfaction with medication. It produces thethree scores: effectiveness, convenience, and overall satisfaction scores. Each score hasconsists of 3three questions, totalling nine questions in all, with each score on a scale from 0 to 100, where higher scores indicate better satisfaction to give 9 in total, with each score on the scale with higher scores better.

EQ-5D-5LEuroQoL 5-Dimension 5-LevelGSEGeneral Self-EfficacyICIQ-LTCqolInternational Consultation on Incontinence Modular Questionnaire-LTC QoLS-CAUTIsymptomatic catheter-associated urinary tract infection

### Outcomes

The follow-up of participants varied from 12 to 24 months due to early closure of the study. A CONSORT diagram is provided (figure 1), which shows adherence to washouts. In the saline group, one participant changed to acidic washout and seven stopped washouts, while in the acidic group, two changed to saline and three stopped washouts. The three changes were all recommendations by the clinical team, while three participants stopped because they were unable to perform the washout, and six stopped for various medical reasons. In the control group, participants experienced a mean of one catheter blockage per month; in the acidic and saline washout groups, the rates were lower: 0.73 and 0.34, respectively. In the washout groups, the rate of LTC blockages requiring treatment was approximately 10 blockages per 1000 catheter days, while in the control group, the rate was approximately 21 per 1000 catheter days (table 2). The IRR favours the washout groups (0.65 (97.5% CI 0.24 to 1.77); p=0.33 and 0.59 (97.5% CI 0.22 to 1.63); p=0.25 for saline and acidic washouts, respectively), although not statistically significant. When the two washout groups are combined in a post hoc analysis (table 2), the IRR was 0.62 (97.5% CI 0.26 to 1.49; p=0.22).

**Table 2:** Blockage requiring treatment (primary outcome) and S-CAUTI

	Saline washouts (n=26)	Acidic washouts (n=27)	Either washout (n=53)	Control (n=27)
Participants providing follow-up data	25	27	52	26
Total months of follow-up	387	409	796	420
Catheterisation duration (days) (mean (SD))	468 (182)	459 (191)	463 (185)	492 (167)
Total number of blockages requiring treatment	105	115	220	236
Blockages requiring treatment (rate per 1000 catheter days) (mean (SD))	9.96 (14.48)	10.53 (15.77)	10.25 (15.02)	20.92 (27.77)
IRR (97.5% CI); p compared with control	0.65 (0.24 to 1.77); 0.33	0.59 (0.22 to 1.63); 0.25	0.62 (0.26 to 1.49); 0.22	
Total instances of S-CAUTI	37	81	118	98
S-CAUTI (rate per 1000 catheter days) (mean (SD))	3.71 (8.45)	6.72 (7.10)	5.27 (7.85)	8.05 (11.29)
IRR (97.5% CI); p compared with control	0.40 (0.20 to 0.80); 0.003	0.98 (0.54 to 1.78); 0.93	0.69 (0.39 to 1.23); 0.14	

IRRincidence rate ratioS-CAUTIsymptomatic catheter-associated urinary tract infection

In the control group, the S-CAUTI rate was eight episodes per 1000 catheter days. In the acidic washout group, the rate was slightly lower at 6.72 per 1000 catheter days, resulting in an IRR of 0.98 (97.5% CI 0.54 to 1.78); p=0.93. In the saline washout group, the S-CAUTI rate was significantly lower at 3.71 per 1000 catheter days, with an IRR of 0.40 (97.5% 0.20 to 0.80); p=0.003.

Online supplemental table S1 shows a sensitivity analysis adjusting for additional factors, which had potential imbalance between groups at baseline. This analysis was consistent with the main analysis and showed that weekly prophylactic LTC washouts reduced LTC blockages requiring intervention and S-CAUTI.

The mean bladder spasm days per month was similar in the washout groups at 3.48 and 3.23 and slightly higher in the control group at 4.38 (table 3).

**Table 3:** Secondary outcomes

	Saline washouts(n=26)	Acidic washouts(n=27)	Control(n=27)
Any catheter blockage (mean per month)	0.34 (0.45)	0.73 (1.84)	1.00 (1.97)
Bladder spasm (mean days per month)	3.5 (5.7)	3.2 (5.9)	4.4 (6.5)
Urine retention (mean days per month)	0.22 (0.45)	0.18 (0.38)	0.37 (0.57)
Blood in urine (mean days per month)	0.25 (0.51)	1.8 (3.8)	1.2 (1.8)
Pus in urine (mean days per month)	1.7 (5.6)	1.3 (4.0)	0.84 (3.3)
Urine leakage (mean days per month)	5.9 (8.7)	4.4 (7.7)	2.0 (6.0)
Catheter kinks (mean instances per month)	0.20 (0.50)	0.051 (0.11)	0.12 (0.31)
Routine catheter changes (mean number per month)	0.34 (0.22)	0.33 (0.23)	0.36 (0.23)
Regular/Preventative washouts (mean number per month)	3.1 (1.4)	3.9 (2.3)	2.6 (6.5)
Treatment of LTC-related AEs			
Hospital visits (mean number per month)	0.0067 (0.024)	0.034 (0.076)	0.051 (0.18)
Primary care visits* (mean number per month)	0.56 (0.41)	0.77 (0.47)	0.92 (0.67)
GP home visits (mean number per month)	0.014 (0.038)	0.031 (0.066)	0.0019 (0.0098)
GP surgery visits (mean number per month)	0.046 (0.12)	0.067 (0.11)	0.11 (0.17)
Nurse home visits (mean number per month)	0.49 (0.36)	0.58 (0.44)	0.72 (0.71)
Nurse practice visits (mean number per month)	0.0087 (0.026)	0.10 (0.24)	0.089 (0.13)
Complication managed by self or informal carer (mean number per month)	0.45 (0.78)	0.62 (1.74)	0.74 (1.59)

The summary statistic in the cells is the mean and standard deviationSD.

* Primary care visits are GP home or surgery visits or nurse home or practice visits.

AEadverse effectGPgeneral practitionerLTClong-term catheter

Patient-reported blood in urine was lowest for those receiving a saline washout and highest for those on an acidic washout. Patient-reported pus in urine was higher than control for both washout groups. Instances of urine leakage were similar for all three groups but both washout groups had a higher mean number of days than the control group.

LTC-related AEs were predominantly managed by the individual/their carer or by a nurse home visit.

The number of participants experiencing other AEs are generally small (table 4).

**Table 4:** Other adverse events

	Saline washouts (n=26)	Acidic washouts (n=27)	Control (n=27)
Any adverse event	9/26 (35%)	11/27 (41%)	12/27 (44%)
Bladder stones	0/25 (0%)	2/27 (7.4%)	4/26 (15%)
Long-term catheterisation discontinuation	3/25 (12%)	2/27 (7.4%)	1/26 (3.8%)
Epididymitis	0/26 (0%)	1/27 (3.7%)	0/27 (0%)
Urosepsis	0/26 (0%)	0/27 (0%)	1/27 (3.7%)
Pyelonephritis	0/26 (0%)	1/27 (3.7%)	0/27 (0%)
Pain at catheter site	1/25 (4.0%)	2/27 (7.4%)	2/26 (7.7%)
Skin irritation/penile trauma at catheter site	2/25 (8.0%)	1/27 (3.7%)	4/26 (15%)
Bleeding or discharge at catheter site	5/25 (20%)	4/27 (15%)	4/26 (15%)
Granulation problems	2/25 (8.0%)	4/27 (15%)	0/26 (0%)
Sepsis/Pneumonia	0/26 (0%)	1/27 (3.7%)	0/27 (0%)
Cause of death certified as myocardial infarction secondary to congestive cardiac failure and cardiomyopathy	0/26 (0%)	0/27 (0%)	1/27 (3.7%)
Death due to (1a) urosepsis, (1b) prostate cancer, (2) type 2 diabetes mellitus, ischaemic heart disease	1/26 (3.8%)	0/27 (0%)	0/27 (0%)
Death due to metastatic breast cancer	0/26 (0%)	0/27 (0%)	1/27 (3.7%)

The summary in the cells is theconsists of count and percentage.

All instances of granulation are from participants using a suprapubic catheter at the time of the event. All patients reporting skin irritation had a suprapubic catheter at the time. One participant in the control group reported penile trauma, and changedswitched from a urethral catheter to a suprapubic catheter and did not report further trauma or irritation.

Table 5 shows the participant-reported QoL outcomes throughout the study. Participants in both washout groups had better scores in EQ-5D-5L and ICECAP-A (adult version) than the control group indicating better QoL, and better impact on day-to-day activities. None, however, are statistically significant. On the GSE Scale, those in the acidic washout group appeared to be better but the difference again was not significant.

**Table 5:** Quality of life outcomes

	Saline washouts (n=26)	Acidic washouts (n=27)	Control (n=27)
EQ-5D-5L*			
Baseline	0.368 (0.405); (n=25)	0.365 (0.359); (n=26)	0.348 (0.373); (n=27)
6 months	0.356 (0.513); (n=18)	0.335 (0.313); (n=20)	0.270 (0.348); (n=22)
12 months	0.386 (0.430); (n=18)	0.412 (0.321); (n=17)	0.339 (0.414); (n=21)
18 months	0.493 (0.403); (n=10)	0.302 (0.453); (n=7)	0.139 (0.264); (n=8)
24 months	0.349 (0.414); (n=4)	0.621 (0.339); (n=3)	−0.077 (0.082); (n=4)
Exit	0.445 (0.541); (n=6)	0.327 (0.491); (n=4)	0.229 (0.211); (n=4)
Effect size compared with control	0.056 (−0.022 to 0.134); 0.11	0.053 (−0.024 to 0.131); 0.12	
GSE Scale†			
Baseline	29.1 (9.1); (n=25)	29.4 (5.7); (n=27)	27.8 (7.6); (n=27)
6 months	27.7 (9.3); (n=19)	27.6 (6.0); (n=20)	26.8 (8.5); (n=23)
12 months	27.4 (9.7); (n=18)	29.2 (5.5); (n=18)	25.1 (7.5); (n=21)
18 months	28.3 (7.7); (n=9)	29.3 (6.0); (n=8)	28.3 (3.6); (n=9)
24 months	28.3 (3.3); (n=4)	30.4 (4.9); (n=4)	27.3 (7.4); (n=4)
Effect size compared with control	0.9 (−1.5 to 3.2); 0.40	2.2 (−0.1 to 4.5); 0.030	
ICECAP-A‡			
Baseline	0.551 (0.216); (n=10)	0.487 (0.223); (n=11)	0.496 (0.218); (n=9)
6 months	0.671 (0.176); (n=8)	0.592 (0.256); (n=10)	0.620 (0.200); (n=8)
12 months	0.606 (0.233); (n=7)	0.450 (0.282); (n=7)	0.611 (0.146); (n=7)
18 months	0.849 (0.000); (n=2)	0.246 (0.349); (n=2)	0.669 (0.203); (n=4)
24 months	0.766 (0.117); (n=2)	0.304 (0.281); (n=3)	0.486 (0.137); (n=2)
Effect size compared with control	−0.076 (−0.221 to 0.068); 0.24	−0.086 (−0.214 to 0.042); 0.13	
ICECAP-O‡			
Baseline	0.488 (0.320); (n=15)	0.601 (0.206); (n=14)	0.669 (0.204); (n=15)
6 months	0.554 (0.268); (n=12)	0.657 (0.227); (n=11)	0.673 (0.241); (n=15)
12 months	0.569 (0.329); (n=11)	0.611 (0.239); (n=9)	0.707 (0.161); (n=13)
18 months	0.511 (0.239); (n=7)	0.614 (0.331); (n=6)	0.666 (0.230); (n=5)
24 months	0.637 (0.078); (n=2)	0.940 (n/a); (n=1)	0.641 (0.219); (n=2)
Effect size compared with control	0.036 (−0.069 to 0.142); 0.44	−0.038 (−0.145 to 0.070); 0.43	
ICIQ-LTC function and concern§			
Baseline	18.3 (9.1); (n=26)	17.3 (9.7); (n=26)	19.1 (9.0); (n=27)
6 months	15.6 (10.1); (n=19)	16.4 (10.2); (n=19)	19.8 (9.6); (n=23)
12 months	12.5 (6.9); (n=15)	18.1 (11.6); (n=15)	17.9 (10.7); (n=20)
18 months	11.9 (5.5); (n=7)	12.3 (7.5); (n=7)	14.2 (12.5); (n=6)
24 months	9.3 (3.3); (n=4)	19.5 (4.4); (n=4)	18.5 (10.0); (n=4)
Effect size compared with control	−1.2 (-4.1 to 1.7); 0.34	0.7 (-2.2 to 3.5); 0.60	
ICIQ-LTC lifestyle§			
Baseline	6.7 (3.4); (n=24)	8.1 (3.3); (n=27)	7.6 (2.9); (n=27)
6 months	7.4 (3.8); (n=19)	7.6 (4.0); (n=17)	8.4 (3.2); (n=21)
12 months	7.8 (4.3); (n=16)	8.1 (3.7); (n=14)	8.4 (3.6); (n=20)
18 months	7.0 (2.6); (n=8)	10.0 (3.5); (n=7)	7.3 (3.4); (n=6)
24 months	7.5 (2.1); (n=2)	8.8 (4.2); (n=4)	5.3 (2.9); (n=4)
Effect size compared with control	−0.1 (−1.6 to 1.4); 0.90	−0.4 (−1.9 to 1.2); 0.60	
Treatment Satisfaction Questionnaire			
Effectiveness¶			
6 months	67.0 (27.9); (n=17)	67.6 (31.3); (n=18)	
12 months	74.2 (30.5); (n=14)	71.8 (18.9); (n=14)	
18 months	83.3 (22.9); (n=5)	77.8 (21.2); (n=5)	
24 months	83.3 (23.6); (n=2)	77.8 (25.5); (n=3)	
Convenience¶			
6 months	82.0 (15.3); (n=17)	73.8 (23.3); (n=18)	
12 months	89.7 (11.3); (n=14)	77.0 (18.9); (n=14)	
18 months	90.7 (13.5); (n=6)	80.0 (18.7); (n=5)	
24 months	91.7 (3.9); (n=2)	74.1 (8.5); (n=3)	
Overall satisfaction¶			
6 months	76.1 (22.7); (n=17)	78.2 (27.7); (n=17)	
12 months	86.7 (20.2); (n=14)	73.0 (29.5); (n=14)	
18 months	88.1 (22.9); (n=6)	84.3 (27.4); (n=5)	
24 months	75.0 (15.2); (n=2)	69.0 (28.9); (n=3)	

The EQ-5D-5L exit questionnaire was for participants who exited the study early or were not at a notional follow-up point when the study ended.

All effect sizes come fromare derived from a mixed effects linear regression, including fixed effects for the two treatment groups, gender, age band, previous blockage, previous S-CAUTI and baseline measure of the outcome. Dummy variables are also included for the timepoint when theat which follow-up is completed. Random effects (intercepts) are included for region and participant to allowaccount for repeated measures acrossover time. The summary statistics are the mean, standard deviationSD, and count, and the effects sizes arepresented as the adjusted mean difference, 97.5% confidence intervalCI and p--value.

* The EQ-5D-5L is a generic QoL measure. It has 5five questions and is on thea scale from −0.594 to 1, with higher scores indicating better QoL.

† The general self-efficacy (GSE) sScale assesses ability to cope with daily life. It has 10 questions and scores are betweenrange from 10 andto 40, with higher scores indicating better outcomes.

‡ The ICECAP-A and ICEPOP-O measure capability in adults and older people, respectively. Both haveconsist of 5five questions and are on thea scale from 0 to 1, with higher scores betterindicating better outcomes.

§ The ICIQ long-term catheterisation quality of life questionnaireLong-Term Catheterisation Quality of Life Questionnaire is a specific quality of life measure. It produces the function and concern score and the lifestyle score. The function and concern score hasconsists of 10 questions and is on thea scale from 0 to 42. The lifestyle score hasconsists of 3three questions and is on thea scale from 3 to 15. For both scores, higher values indicate worse outcomesFor both higher scores are worse.

¶ The treatment satisfaction questionnaireTreatment Satisfaction Questionnaire assesses satisfaction with medication. It produces three scores:the effectiveness, convenience, and overall satisfaction scores. Each score hasconsists of 3three questions to give 9 in totalfor a total of nine questions, with each score on thea scale from 0 to 100, with higher scores betterwhere higher scores indicate better satisfaction.

EQ-5D-5LEuroQoL 5-Dimension 5-LevelGSEGeneral Self-EfficacyICIQ-LTCInternational Consultation on Incontinence Modular Questionnaire-LTCQoLquality of life

Participants either received the ICECAP-A for adults or ICECAP-O for the older population. This had the effect of splitting the trial population and increasing the uncertainty around the effect sizes. For the ICIQ-LTCqol scores, there was little evidence of any difference between the groups.

The Treatment Satisfaction Questionnaire, completed only by those in the washout groups, suggests that participants in the saline group were more satisfied.

The Time and Travel Questionnaire was completed at 18 months by 24 participants. Online supplemental table S2 summarises the distance travelled for appointments and admissions, the cost of journeys and the total time taken.

## Discussion

The CATHETER II RCT was terminated early primarily due to the impact of the COVID-19 pandemic. The vast majority of NHS research capacity in the UK, especially in primary care, was directed to COVID-19 research with QoL research, including CATHETER II, categorised as lower priority. Four months after starting CATHETER II, recruitment was temporarily paused and never recovered satisfactorily due to limited research capacity in primary and secondary care. The funder elected for early termination of the study. Consequently, our results are limited by the significantly smaller sample size (n=80) than originally planned (n=600).

However, the CATHETER II results indicated a favourable trend for lower rates of LTC blockages in both the prophylactic washouts groups, although not statistically significant. The rate of LTC blockages per 1000 catheter days requiring treatment were 9.96, 10.53 and 20.92 in the saline, acidic and control groups, respectively. The IRR favours the washout groups (0.65 (97.5% CI 0.24 to 1.77); p=0.334 and 0.59 (97.5% CI 0.22 to 1.63); p=0.25 for saline and acidic, respectively), but neither reach statistical significance most likely due to the small sample size. Gage *et al*^5^ indicated that hospital resource use accounted for almost half of health services costs, mainly due to unplanned hospital admission for LTC blockage or S-CAUTI. Reduction in LTC blockage is likely to reduce the healthcare costs, as fewer emergency treatments will be required. In CATHETER II, there were fewer visits to and by healthcare professionals in the washout groups. However, we were unable to perform a full health economic analysis due to the early termination and, consequently, the small sample size.

Catheter blockages impact up to 50% of individuals living with LTC, leading to discomfort and emotional distress.^23^ Shepherd *et al*^7^ conducted a Cochrane systematic review comparing washout policies in patients with LTC. They summarised results of seven RCTs, including 349 participants, of which 217 participants completed the trials. The authors concluded that evidence on the benefits and risks of various washout policies was limited and generally of low quality. Moore *et al*^24^ conducted a three-arm RCT using saline or acidic solutions and compared it with standard care with no washout. They reported results from 53 participants and found insufficient evidence to determine whether prophylactic LTC washout with saline or acidic solution was more effective than standard care without washout in preventing blockages. Muncie *et al*^25^ (n=32) provided data on the mean catheter replacement rate per 100 days of catheterisation. They reported the mean was 5.5 catheters replaced for the saline washout period and 4.7 catheters replaced for no washout periods, indicating no significant impact on the incidence of the total number of catheter replacements. The British Association of Urological Surgeons and Nurses consensus document indicates that prophylactic bladder washouts or catheter maintenance solutions can be employed to minimise the risk of catheter blockages in patients living with LTC.^26^ In CATHETER II, the observed trends in reduced LTC blockage rates in the washout groups, despite the lack of statistical significance, suggest a potential benefit of prophylactic washouts in preventing LTC blockages. Hence, we propose further research with larger sample sizes to validate these findings. This can be best achieved by an international RCT in countries with similar healthcare systems.

S-CAUTI is the main safety issue with prophylactic LTC washouts and was the concern stated in the National Institute for Health and Care Excellence guideline development group as potential harm and one of the main reasons for not recommending prophylactic LTC washouts.^1 27^ The Cochrane review^7^ included four trials comparing saline or acidic washouts with no washout. There was insufficient evidence from these trials and the Cochrane review could not draw a conclusion if there was an effect on S-CAUTI incidence or catheterisation duration. It is therefore reassuring to see in CATHETER II, despite the small sample size, the S-CAUTI rate is significantly lower at 3.71 per 1000 catheter days in the saline washout group compared with 8 per 1000 catheter days in the standard LTC care-only group (IRR 0.40 (97.5% CI 0.20 to 0.80); p=0.003). There are also lower rates of S-CAUTI in the acidic washout group at 6.72 per 1000 catheter days (IRR 0.98 (97.5% CI 0.54 to 1.78); p=0.926), although not reaching statistical significance. Moore *et al*^24^ (n=32) reported no incidence of S-CAUTI in their trial participants. Self-reported urinary tract infections, however, were reported in each group (citric acid 5/24, saline 2/18, no washout 3/23).

In CATHETER II, the mean monthly occurrence of bladder spasms was comparable between the washout groups and slightly higher in the control group. All three groups had <1 day of urine retention per month. In the Cochrane review,^7^ only one trial reported results of bladder spasm; saline 0/29 participants, acetic acid 1/30 participants, neomycin-polymyxin 2/30 participants.^28^

Participants receiving a saline washout experienced fewer episodes of blood in urine compared with the control group, while those on an acidic washout had higher occurrences.

Moore *et al*^24^ presented findings from urine dipstick testing, revealing a consistent presence of blood in the urine for all participants, regardless of their assigned groups.

Washout groups had more days of leakage (catheter bypass) on average than the control. Muncie *et al*^25^ in their crossover trial reported 32 events of urine leakage, 11/32 in the saline washout period and 21/32 in no washout period. Catheter kinks were rare in all groups. Although some differences were observed between the washout groups and the control group in terms of self-reported blood and pus in urine and pus in urine, the incidence of other events was similar.

The incidence of AEs among participants in all groups was low. Bleeding or discharge at the catheter site shows comparable rates across all three groups. Granulation tissue formation occurred only in the suprapubic catheter sites and are exclusively noted in the washout groups, with two occurrences in the saline group and four in the acidic group. Most complications were primarily handled by either the individual themselves, their carer or through a nurse’s home visit.

In this trial, participants performed prophylactic washouts with self-care and minimal dependence on healthcare resources. The participants were provided with video training that was proven to be effective with only four participants stopping the intervention for inability to perform washouts. Results of the TSQM questionnaire showed relatively high scores for convenience, effectiveness and overall satisfaction in both the LTC washout groups. There are no other studies in the literature that made similar comparisons.

Acceptability of prophylactic LTC washouts and the self-care programme was further confirmed in the embedded qualitative study (reported in a separate publication).

The Cochrane review^7^ noted that none of the RCTs assessed patients’ acceptability and/or impact on QoL and recommended these outcomes should be assessed in future RCTs. In CATHETER II, participants in both washout groups showed higher EQ-5D-5L scores than the control group, indicating potential for greater improvement in QoL, although not statistically significant. There was little evidence of differences between groups in terms of ICIQ-LTC scores.

### Strengths and limitations

CATHETER II is a robustly designed pragmatic RCT abiding by the principles and recommendations of the CONSORT statement. The RCT included an embedded qualitative study highlighting the views and experience of patients and healthcare professionals (reported separately). We assessed a comprehensive list of outcomes, which are relevant for patients, healthcare professionals, guideline developers and other stakeholders’ decision-making. Women constituted approximately 50% of the study population and were balanced between groups confirming generalisability of our results.

Despite being the largest reported RCT on this topic, a significant limitation is the small sample size; hence, the trial was underpowered to detect the 25% reduction in catheter blockage it aimed to demonstrate. Hence, the results cannot be used on their own to implement change in current clinical practice. However, they can be part of a wider meta-analyses in this field. A large adequately powered RCT may be required in the future, however its feasibility may be doubtful unless international collaboration is utilised.

### Conclusions

The early closure and small sample size of the CATHETER II RCT limits our ability to determine the comparative effectiveness between saline or acidic catheter washout solutions in addition to standard LTC care compared with standard LTC care only. However, the results are favourable, although not statistically significant, for lower rates of LTC blockages without a rise in S-CAUTI when employing prophylactic LTC washouts. We therefore recommend an international RCT to ascertain the clinical and cost-effectiveness of LTC washouts.

## supplementary material

10.1136/bmjopen-2024-087203 online supplemental file 2

## Data Availability

Data are available on reasonable request.
