# Supplementary material for: CATHETER II: a randomised controlled trial comparing the clinical effectiveness of various washout policies versus no washout policy in preventing catheter-associated complications in adults living with long-term catheters
Source: BMJ Open. 2024 Dec 2;14(12):e087203. doi: 10.1136/bmjopen-2024-087203 (PMC11624782; doi:10.1136/bmjopen-2024-087203)
Supplement: online supplemental file 2 [file bmjopen-14-12-s001.docx]

**SUPPLEMENTARY MATERIALS**

**Table S1** Sensitivity analyses

|  | Saline washouts  (n=26) | Acidic washouts  (n=27) | Either washout  (n=53) | Control  (n=27) |
| --- | --- | --- | --- | --- |
| Participants providing follow-up data | 25 | 27 | 52 | 26 |
| Total months of follow-up | 387 | 409 | 796 | 420 |
| Mean catheterisation duration (days) | 468(182) | 459(191) | 463(185) | 492(167) |
| Blockages requiring treatment (rate per 1000 catheter days) | 9.96(14.48) | 10.53(15.77) | 10.25(15.02) | 20.92(27.77) |
| IRR compared to control | 0.65(0.24 to 1.77);0.33 | 0.59(0.22 to 1.63);0.25 | 0.62(0.26 to 1.49);0.22 |  |
| Sensitivity analysis | 0.85(0.29 to 2.49);0.74 | 0.68(0.24 to 1.94);0.41 | 0.76(0.30 to 1.95);0.52 |  |
|  |  |  |  |  |
| S-CAUTI (rate per 1000 catheter days) | 3.71(8.45) | 6.72(7.10) | 5.27(7.85) | 8.05(11.29) |
| IRR compared to control | 0.40(0.20 to 0.80);0.003 | 0.98(0.54 to 1.78);0.93 | 0.69(0.39 to 1.23);0.14 |  |
| Sensitivity analysis | 0.30(0.16 to 0.56);<0.001 | 0.66(0.38 to 1.15);0.09 | 0.47(0.28 to 0.80);0.001 |  |

Due to the early closure of the trial and small sample size there was potential imbalance at baseline that would have been eliminated if 200 participants had been randomised to each group. Therefore, an additional analysis was conducted of blockage requiring intervention and S-CAUTI.

The results of the sensitivity analysis show the IRR for the primary outcome are closer to 1. This indicates either washout reduces the number of blockages requiring treatment, but the effects are not as strong. The sensitivity analysis of infections requiring antibiotics show lower IRR from both saline and acidic washout. There is a strong suggestion that both washouts reduce S-CAUTI. Compared to the trial analysis the effect from acidic washout on S-CAUTI is stronger.

**Table S2** Time and travel data

|  | Saline washouts  (n=26) | Acidic washouts  (n=27) | Control  (n=27) |
| --- | --- | --- | --- |
| Participants completed questionnaire | 8 | 7 | 9 |
| Travel to outpatient consultation |  |  |  |
| distance (miles) | 15.0(n/a);[N=1] | 16.0(12.5);[N=3] | 13.6(6.5);[N=7] |
| cost (£) | 0.00(0.00);[N=2] | 0.00(0.00);[N=3] | 1.11(1.97);[N=7] |
| Total outpatient time (hours) | 5.33(n/a);[N=1] | 3.33(1.53);[N=3] | 3.09(1.80);[N=7] |
| Travel to GP appointment |  |  |  |
| distance (miles) |  | 1.0(n/a);[N=1] | 3.3(1.9);[N=6] |
| cost (£) |  | 0.00(n/a);[N=1] | 0.00(0.00);[N=6] |
| Total time for GP appointment (hours) |  | 1.00(n/a);[N=1] | 1.56(1.12);[N=6] |
| Travel to hospital admission |  |  |  |
| distance (miles) |  | 8.0(0.0);[N=2] | 13.8(1.5);[N=4] |
| cost (£) |  | 0.00(n/a);[N=1] | 1.00(1.73);[N=3] |
| Total time for hospital admission(days) |  | 6.00(n/a);[N=1] | 6.76(2.06);[N=4] |

The summary statistic in the cells is the mean, standard deviation, and count.
