# Supplementary material for: CATHETER II: a randomised controlled trial comparing the clinical effectiveness of various washout policies versus no washout policy in preventing catheter-associated complications in adults living with long-term catheters
Source: BMJ Open. 2024 Dec 2;14(12):e087203. doi: 10.1136/bmjopen-2024-087203 (PMC11624782; doi:10.1136/bmjopen-2024-087203)
Supplement: Supplementary file 2 [file bmjopen-2024-087203.reviewer_comments.pdf]

PEER REVIEW HISTORY

BMJ Open publishes all reviews undertaken for accepted manuscripts. Reviewers are asked to complete a checklist review form and are provided with free text boxes to elaborate on their assessment. These free text comments are reproduced below.

ARTICLE DETAILS

Title (Provisional)

CATHETER II, a randomised controlled trial comparing the clinical effectiveness of various washout policies versus no washout policy in preventing catheter associated complications in adults living with long term catheters

Authors

Abdel-fattah, Mohamed; Omar, Muhammad Imran; Johnson, Diana; Cooper, David; Constable, Lynda; Tripathee, Dr Sheela; Maclennan, Sara J; Dimitropoulos, Konstantinos; Evans, Suzanne; Hashim, Hashim; Kilonzo, M; Larcombe, James; Little, Paul; Murchie, Peter; Myint, Phyo Kyaw; NDow, James; Paterson, Catherine; Powell, Karen; Scotland, Graham; Thiruchelvam, Nikesh; Young, Amanda; Cotton, Seonaidh; Norrie, John; MacLennan, Graeme

---

VERSION 1 - REVIEW

**Reviewer**	**1**
**Name**	**Jyani, Gaurav**
**Affiliation**	**Post Graduate Institute of Medical Education and Research, Department of Community Medicine and School of Public Health**
**Date**	**31-May-2024**
**COI**	**There are no competing interests.**

Thank you for inviting me to review this paper. I would like to sincerely appreciate the authors for their hard work in conducting this study and preparing this manuscript. This study attempted to evaluate clinical effectiveness, patient acceptability, satisfaction, and safety of using weekly prophylactic normal saline or citric acid washouts as compared to standard long term catheter care. It is indeed a very important topic, but the sample size of the study is too small to generate any conclusive evidence. Given the significant limitations, the manuscript may not provide conclusive evidence but contributes valuable preliminary data. If the journal's criteria are stringent, the manuscript may not meet the threshold for publication. However, it provides a basis for future research and could be considered for publication in a journal that values preliminary findings. Here are my comments:

1. Ensure clarity and consistency in reporting results, avoiding any language that might imply significance where there is none. The authors have mentioned that the various outcome measures were different among the groups, albeit not statistically significant. My suggestion is that if difference is not statistically significant, it should not be worded as a ‘difference’ favoring one group, rather should be mentioned as ‘no difference was observed among the groups’, as it may have obtained by chance. Therefore, it is suggested to reframe the description on differences throughout the manuscript to emphasize non-significance: "No significant differences were observed among the groups, indicating that the observed trends could be due to chance."

2. The study's small sample size significantly limits its power to detect meaningful differences. This is a critical limitation and should be a major consideration in interpreting the results.

3. Variability in how washouts are performed could affect outcomes. Provide details on how washout procedures were standardized. What statistical approaches were used to factor in the variability caused by difference in the process of performing washouts?

4. The title of the manuscript mentions 'cost-effectiveness,' which may be misleading given that the economic evaluation was not performed due to the early termination of the study. The term 'cost-effectiveness' suggests that the study includes a detailed analysis of the economic implications of the interventions, comparing costs and outcomes. However, without this analysis in the manuscript, we cannot draw any conclusions about the economic viability of the interventions. It is suggested to edit the title of the paper accordingly, although it is understandable that it currently flows from the title of the trial wherein the intention to perform an economic analysis was part of the original study design, possibly influencing the title. However, in its current state, the manuscript does not provide the necessary data to support claims of cost-effectiveness.

5. Emphasizing non-significance is crucial. The manuscript should avoid suggesting clinical implications based on non-significant findings. The conclusion could more explicitly state that the current study's results are not sufficient to change clinical practice. More emphasis could be placed on the need for larger, adequately powered studies.

6. On page 10, while describing outcomes, may use the standard terminology, i.e., quality adjusted life year (QALY), not QoLY. However, as the economic evaluation was not performed, it is suggested to remove the mention of the primary health economic outcome from the methods.

7. While using EQ-5D-5L, wasn’t the information on Visual Analog Scale (VAS) was collected? The VAS is an integral part of the EQ-5D, and should always be administered alongside EQ-5D descriptive system.

**Reviewer**	**2**
**Name**	**Laan, Bart**
**Affiliation**	**Amsterdam UMC - Locatie AMC, Internal Medicine, Infectious Diseases**
**Date**	**01-Jun-2024**
**COI**	**No competing interest**

Thank you for the opportunity to review this pragmatic randomized controlled trial investigating whether prophylactic catheter washout prevents blockage. It is unfortunate that the study had to stop because of the COVID pandemic. Although it was not possible to blind patients and the sample size was limited, this study is valuable.

Could the authors clarify why they chose (normal saline and) citric acid for the washouts?

Did the authors make any changes to the manuscript compared to the protocol besides the sample size? Where is the cost-effectiveness analysis?

Why did the authors use 60 ml of citric acid and 100 ml of NaCl 0.9%?

What was the definition of catheter blockage? Was it patient-reported, and if so, did it include only cases where catheter change was necessary or also mild/temporary blockages?

Could it be that the outcome is influenced by the fact that the saline group changed the catheter less frequently?

Additionally, I would have appreciated a baseline and subgroup analysis by catheter type/route.

The absence of patient blinding is a significant limitation that warrants attention in the discussion.

It is unfortunate that B. Braun Medical AG commented on the manuscript prior to submission.

This was a well-conducted study; however, due to issues with statistical power, no significant results were obtained.

VERSION 1 - AUTHOR RESPONSE

Reviewer: 1
Dr. Gaurav Jyani, Post Graduate Institute of Medical Education and Research
Comments to the Author:
Thank you for inviting me to review this paper. I would like to sincerely appreciate the authors for their hard work in conducting this study and preparing this manuscript. This study attempted to evaluate clinical effectiveness, patient acceptability, satisfaction, and safety of using weekly prophylactic normal saline or citric acid washouts as compared to standard long term catheter

care. It is indeed a very important topic, but the sample size of the study is too small to generate any conclusive evidence. Given the significant limitations, the manuscript may not provide conclusive evidence but contributes valuable preliminary data. If the journal's criteria are stringent, the manuscript may not meet the threshold for publication. However, it provides a basis for future research and could be considered for publication in a journal that values preliminary findings. Here are my comments:

1. Ensure clarity and consistency in reporting results, avoiding any language that might imply significance where there is none. The authors have mentioned that the various outcome measures were different among the groups, albeit not statistically significant. My suggestion is that if difference is not statistically significant, it should not be worded as a 'difference' favoring one group, rather should be mentioned as 'no difference was observed among the groups', as it may have obtained by chance. Therefore, it is suggested to reframe the description on differences throughout the manuscript to emphasize non-significance: "No significant differences were observed among the groups, indicating that the observed trends could be due to chance."

We are thankful to the reviewer for their comment and respectfully disagree. On the primary outcome there are approximately 20 blockages requiring treatment in the control group and 10 blockages requiring treatment in both of the intervention groups. The incidence rate ratios of 0.65 and 0.59 also suggest that washout is beneficial in comparison to the control. These differences are likely to be clinically important even though not statistically significant. We cannot phrase that there was no difference observed among the groups when there was "a difference" observed. However, it will be correct to phrase that the difference observed was not statistically significant which we use throughout the manuscript including the Results section. We also comment on clinical significance within the Discussion section.

2. The study's small sample size significantly limits its power to detect meaningful differences. This is a critical limitation and should be a major consideration in interpreting the results.

We agree that the trial results are limited by the small sample size and this is acknowledged several times within the manuscript (including the abstract). We specifically highlighted this within the conclusions and as a key limitation within the discussion.

3. Variability in how washouts are performed could affect outcomes. Provide details on how washout procedures were standardized. What statistical approaches were used to factor in the variability caused by difference in the process of performing washouts?

Thanks. It is important to highlight that CATHETER II was a pragmatic RCT to reflect the true clinical practice hence there will certainly be variation in how the washouts are performed. However, as we clearly state in the study protocol (Abdel-fattah *et al.,* 2022) we have ensured the participants and /or their carers received the appropriate training to be able to perform the washouts in line with best clinical practice and the manufacturers guidance. We have now added under "interventions" a clarification regarding training.

Sites were trained by the central trial office in washout best practice. All sites were supplied with the same training materials. Only delegated and trained members of staff could train participants (and their carers, as applicable) to administer washouts in accordance with this training. This training was delivered either remotely or face to face. All participants had access to a freely available online training video in addition to a laminated set of instructions, to support administration of the washouts in accordance with best practice. Sites were instructed to ensure that they were confident the participant (and/or carer) could administer the washouts in accordance with best practice. If additional training was required, this was identified at the initial training visit or subsequently through the regular phone calls with the participant to collect outcome data. Additionally, participants were provided with contact details for the sites should any queries arise. Sites would provide additional training to participants/carers if this need was identified. Adherence and any problems with the weekly washout were documented. All sites were provided with a troubleshooting guide, which had input from urologists, nurses and the manufacturer of the products, to support participants (and/or carer) as required. If a participant was not able to administer the washouts, the participant was withdrawn from the intervention of the study.

Given the pragmatic nature of the study, we did not collect data around potential variability of washout use so are unable to adjust for this in the analysis.

4. The title of the manuscript mentions 'cost-effectiveness,' which may be misleading given that the economic evaluation was not performed due to the early termination of the study. The term 'cost-effectiveness' suggests that the study includes a detailed analysis of the economic implications of the interventions, comparing costs and outcomes. However, without this analysis in the manuscript, we cannot draw any conclusions about the economic viability of the interventions. It is suggested to edit the title of the paper accordingly, although it is understandable that it currently flows from the title of the trial wherein the intention to perform an economic analysis was part of the original study design, possibly influencing the title. However, in its current state, the manuscript does not provide the necessary data to support claims of cost-effectiveness.

Thank you – we have amended the title as per your recommendation.

5. Emphasizing non-significance is crucial. The manuscript should avoid suggesting clinical implications based on non-significant findings. The conclusion could more explicitly state that the current study's results are not sufficient to change clinical practice. More emphasis could be placed on the need for larger, adequately powered studies.

Thanks for your comment: we agree with the reviewer and have amended the manuscript accordingly.

Under limitations in discussion section, we clarified “Despite being the largest reported RCT on this topic, a significant limitation is the small sample size hence the trial was underpowered to detect the 25% reduction in catheter blockage it set out to demonstrate. Hence the results cannot be used on their own to implement change in current clinical practice. However, they

can be part of a wider meta-analyses in this field. A large adequately powered RCT may be required in the future however its feasibility may be doubtful."

We already recommend an RCT in our conclusion as we state: "We therefore recommend an international RCT to ascertain the clinical and cost-effectiveness of LTC washouts."

6. On page 10, while describing outcomes, may use the standard terminology, i.e., quality adjusted life year (QALY), not QoLY. However, as the economic evaluation was not performed, it is suggested to remove the mention of the primary health economic outcome from the methods.

The error on page 10 has now been corrected in the manuscript. Thank you for flagging this.

We understand the reasoning presented for excluding the primary health economic outcome from the methods. However, our reference to the primary health economic outcome is intentional. We aimed to follow the CONSORT guidelines and report the study design and outcomes as per the original intentions of the study with the changes to methods and outcomes following trial commencement noted in the methods section.

7. While using EQ-5D-5L, wasn't the information on Visual Analog Scale (VAS) was collected? The VAS is an integral part of the EQ-5D, and should always be administered alongside EQ-5D descriptive system.

The VAS was used and collected as part of the EQ-5D-5L measure. The licensor validated the use of the paper and electronic versions of the questionnaire used in the study. We can add the VAS data to the manuscript if it would be helpful.

Reviewer: 2
Mr. Bart Laan, Amsterdam UMC - Locatie AMC
Comments to the Author:
Thank you for the opportunity to review this pragmatic randomized controlled trial investigating whether prophylactic catheter washout prevents blockage. It is unfortunate that the study had to stop because of the COVID pandemic. Although it was not possible to blind patients and the sample size was limited, this study is valuable.

1. Could the authors clarify why they chose (normal saline and) citric acid for the washouts?

Several catheter washout policies were used in clinical practice in the UK for the prevention and management of catheter blockage, this included different types of washouts, volumes and frequency of administration.

We sought to compare NaCl 0.9% and citric acid 3% from the outset of the study. The choice of these comparators was agreed following survey of opinion and practice of collaborating units, general practitioners, patients and carers, Bladder Health UK and nurses from BAUN, EAUN and RCN. Their responses showed an overwhelming preference for a three-arm design that included

the two main types and most commonly used washouts (acidic and saline). Additionally, acidic and saline were the most frequently used interventions in the small RCTs included in the Cochrane systematic review on washout policies. Further clarification on this point has been added to the 'Interventions' section of the paper (page 9).

2. Did the authors make any changes to the manuscript compared to the protocol besides the sample size? Where is the cost-effectiveness analysis?

Due to the small sample size, it was not possible to complete the planned subgroup analyses. It was not possible to analyse and report primary (incremental cost per quality adjusted life year) or secondary (time and travel costs) economic outcomes. Health care resource use was presented instead as descriptive analysis. We also included a sensitivity analysis of blockage requiring intervention and S-CAUTI to address potential baseline imbalance as a result of small sample size. These changes are noted in the statistical methods section of the paper on page 13.

The statistical analysis plan noted, due to small sample size, that descriptive statistics may only be provided due to low event rates or lack of variation and that it may be inappropriate to adjust for all minimisation covariates due to low counts for some of the subcategories. We were unable to adjust in the analysis for urine pH due to a lack of variation between participants.

Table 1 (baseline data) and Table 2 (blockage requiring treatment (primary outcome) and S-CAUTI) were disaggregated by gender and included in the supplementary materials (Table S4 and Table S5) to meet the journal requirements for BMJ. We have now removed these tables as they are no longer required.

3. Why did the authors use 60 ml of citric acid and 100 ml of NaCl 0.9%?

We describe the reason for the choice of citric acid and NaCl in the response to comment #1 above. The volumes chosen were primarily determined by which catheter washout products were most commonly used within the UK at the time, to best compare the catheter washout policies in use in the UK. A secondary consideration was product availability and manufacturing timelines within the lifetime of the study. The products were provided by B. Braun Medical AG who confirmed that these volumes were the most commonly used within the UK and could be supplied within the required timeframes. A brief justification of washout choice has been added to the manuscript (page 9).

4. What was the definition of catheter blockage? Was it patient-reported, and if so, did it include only cases where catheter change was necessary or also mild/temporary blockages?

All outcome data was patient reported. For the purposes of the study, "catheter blockage" was defined as one requiring intervention including, but not limited to catheter change. Other interventions included in our definition were unplanned catheter removal, washout performed by the participant / designated person, or required unplanned visits to/from any healthcare

provider, or hospital admission. We have clarified this in the manuscript on page 10.

5. Could it be that the outcome is influenced by the fact that the saline group changed the catheter less frequently?

At baseline a higher proportion of participants changed their catheter at 12-week intervals in the saline group (table 1). However, table 3 shows routine catheter changes during follow up was similar across all three groups. In the sensitivity analysis we included a term to indicate baseline catheter change frequency. The sensitivity analysis still shows saline to be less effective than acidic for reducing blockage and more effective than acidic for reducing infection.

6. Additionally, I would have appreciated a baseline and subgroup analysis by catheter type/route.

Due to the small sample size, it was not possible to conduct subgroup analyses.

7. The absence of patient blinding is a significant limitation that warrants attention in the discussion.

It would not be possible to blind the no prophylactic washout standard LTC care only arm. As for the washout arms, it would not be possible to blind due to the use of two different volumes (100ml for the saline and two sequential applications of 30mls for the citric). Additionally, proprietary washouts in existing packaging were used as a cost-saving measure. It would have been prohibitively expensive to package saline and citric acid identically. The manuscript has been updated to note that it was not possible to blind participants to the intervention (page 9).

8. It is unfortunate that B. Braun Medical AG commented on the manuscript prior to submission.

B. Braun Medical AG supplied the washouts for the study free of charge. The agreement between the study sponsor (University of Aberdeen and NHS Grampian) and B. Braun Medical AG limited the involvement of B Braun Medical AG to the provision of the washouts with no right whatsoever to influence the data or results of the trial by any means. B. Braun Medical AG had no input into any part of the study design or analysis or writing and had no access to the data. As a condition of supply of the washouts, B. Braun Medical AG required the opportunity to review and comment on any manuscript intended for publication, prior to submission. The supply agreement with B. Braun Medical AG is explicit that if B. Braun Medical AG proposed changes to the manuscript, these would be considered if they did not compromise the scientific nature or neutrality of the publication. This has been disclosed on pages 31 and 32 of the manuscript.
The comments received from B. Braun Medical AG for this paper concerned methodological queries about the statistical analysis, crossover, adherence to the intervention, adverse events, cost analysis and typographical errors. In practice, the only changes made to the manuscript as a result of these comments were corrections of typographical errors and inclusion of missing baseline values in Table 5.

9. This was a well-conducted study; however, due to issues with statistical power, no significant results were obtained.

We agree that the study results are limited by the small sample size and this is acknowledged several times within the manuscript (including the abstract). We specifically highlighted this key limitation in the conclusions and within the discussion.

We have further clarified in the strengths and limitations section on page 26 "Despite being the largest reported RCT on this topic, a significant limitation is the small sample size hence the trial was underpowered to detect the 25% reduction in catheter blockage it set out to demonstrate. Hence the results cannot be used on their own to implement change in current clinical practice. However, they can be part of a wider meta-analyses in this field. A large adequately powered RCT may be required in the future, however its feasibility may be doubtful."
